# Translevator perineal hernia; an incidental rare imaging presentation

**DOI:** 10.1259/bjrcr.20220009

**Published:** 2022-06-08

**Authors:** Wendy S Coalter, Salman A Siddiqui

**Affiliations:** 1 Radiology Registrar, Northern Ireland Medical and Dental Training Agency (NIMDTA), Belfast, United Kingdom; 2 Consultant Radiologist, Altnagelvin hospital, Londonderry, United Kingdom

## Abstract

Translevator perineal hernias are rare pelvic floor defects that can be classified based on their anatomical location as either anterior or posterior relative to the transverse perineal muscle. This report describes an unusual clinical and radiological presentation of a posterior perineal hernia in an elderly lady. The case highlights the limitations of CT when findings are not definitive and the role of MRI as a superior problem-solving tool to better delineate the pelvic anatomy, exclude sinister pathology and confirm the diagnosis.

## Case presentation

An 84-year-old lady presented with worsening frailty and unintentional weight loss. No altered bowel habits or tenesmus was reported. Her past medical history included Parkinson’s disease and hysterectomy for a non-cancerous cause. Clinical examination and blood tests were unremarkable.

## Investigations and imaging findings

**Figure 1.**
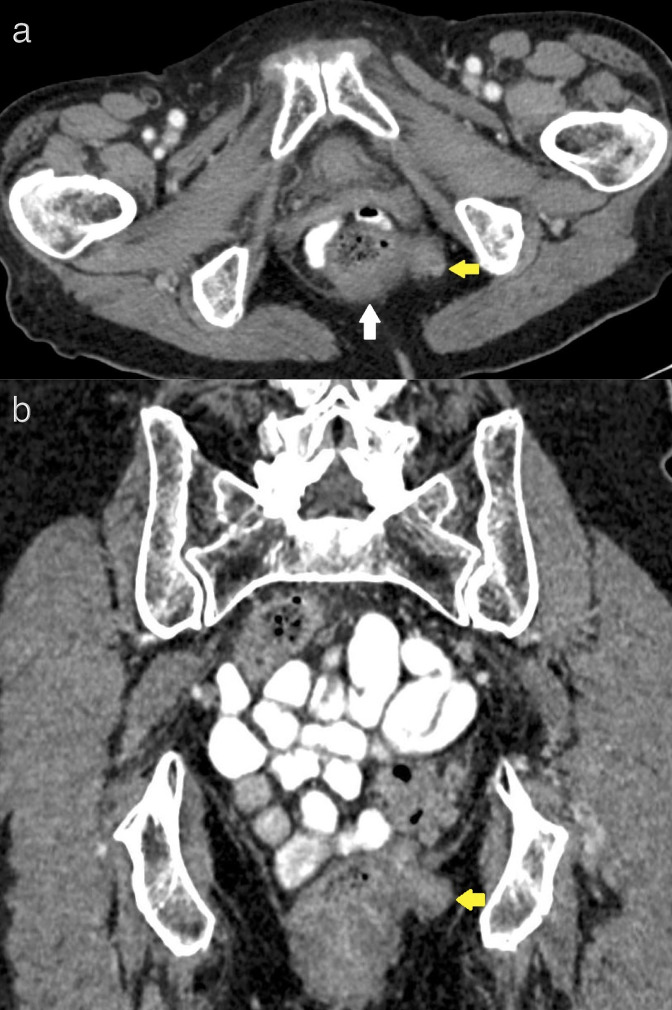
Axial (**a**) and coronal (**b**) contrast-enhanced, portal venous phase CT images through the pelvis at the level of the ischial fossae demonstrate eccentrically thickened left posterolateral rectal wall (white arrow) with an exophytic component (yellow arrow) breaching the meso-rectal fascia and extending into the left ischiorectal fossa at 4 o‘clock position.

**Figure 2.**
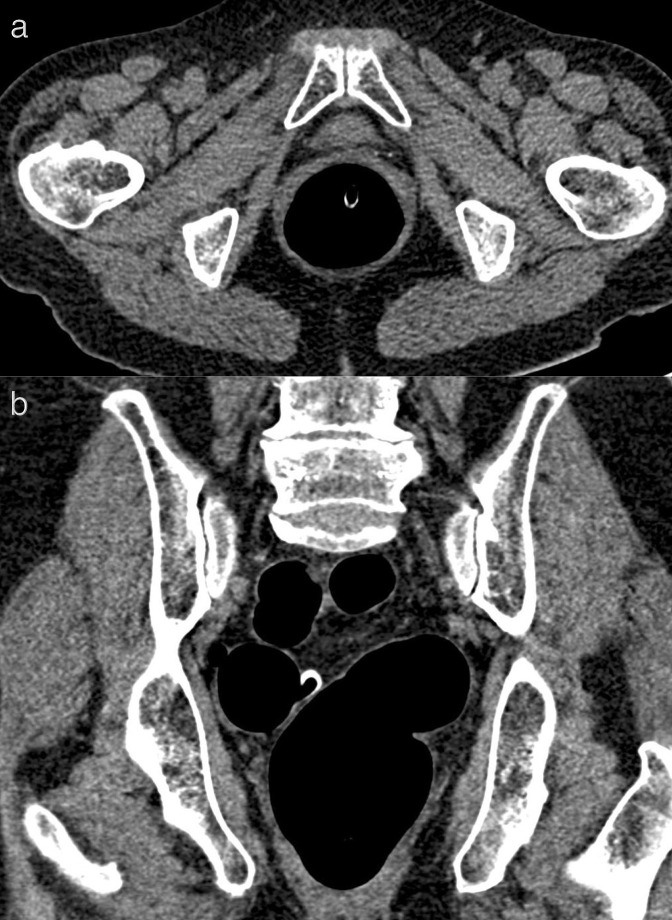
Axial (**a**) and coronal (**b**) image of virtual CT colonoscopy from 2016 showing no rectal or pelvic pathology.

**Figure 3.**
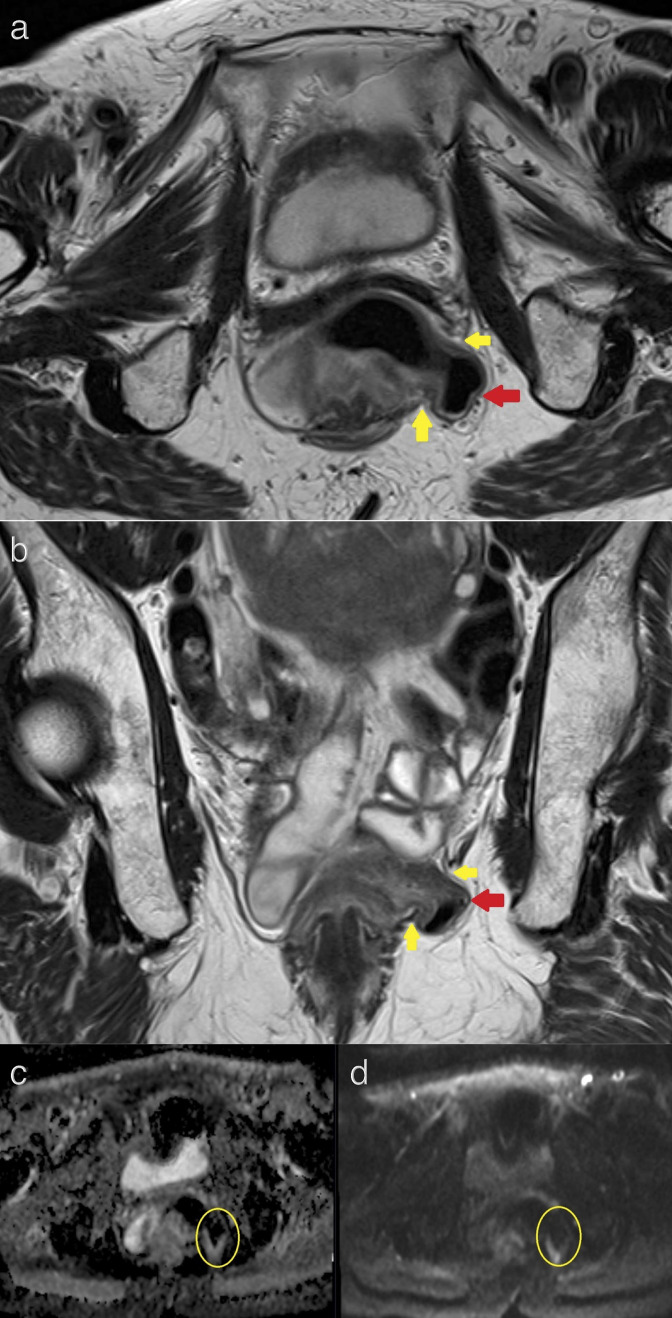
Axial (**a**) and coronal (**b**) *T*_2_-weighted rectal MRI images demonstrating a small Richter type hernia of the left lateral rectal wall (red arrow) through a 22 mm defect in the left levator ani muscle (yellow arrow). No restricted diffusion is noted on ADC (**c**) and DWI (**d**) axial sequences in the region of the perineal hernia (yellow circles).

The patient underwent CT chest, abdomen and pelvis as a part of the initial workup to exclude any sinister pathology. CT showed eccentrically thickened left posterolateral rectal wall with an exophytic component breaching the meso-rectal fascia and extending into the left ischiorectal fossa at 4 o‘clock position raising the concern for a sinister locally advanced rectal mass (Figure 1). However, the lesion did not appear centred on the rectal wall, it was not associated with peripheral fat stranding and metastatic local lymph nodes were not present on CT, favouring a benign aetiology. Retrospective review of virtual CT colonoscopy from 2016 revealed no rectal or pelvic pathology (Figure 2). The patient proceeded for flexible sigmoidoscopy which only revealed a small rectal diverticulum but no mass. Subsequently, MRI rectum was performed which clarified the CT and sigmoidoscopy findings and confirmed a small Richter’s type hernia of the left lateral rectal wall through a 22 mm defect in the left levator ani muscle (Figure 3). No suspicious rectal thickening or mass was identified on MRI. The DWI sequences did not show restriction of diffusivity at the mass level, confirming benignity of the lesion.

## Differential diagnosis

Rectal cancer was the main differential diagnosis in our case which is unique to the already documented benign differential diagnoses in the available literature including pelvic lipomas, fibromas, rectoceles, cystoceles or rectal prolapse.^1^

## Treatment and prognosis

Given the patient’s age, frailty and lack of symptoms, the patient was managed conservatively for this small incidental posterior perineal hernia.

## Discussion

**Figure 4.**
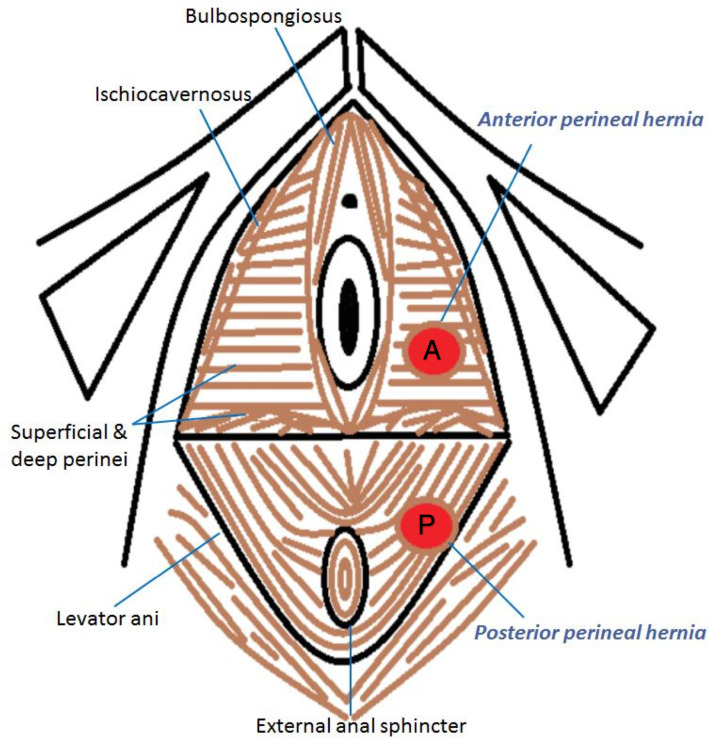
Line diagram showing cross-sectional (axial) anatomy of female perineal musculature. Anterior (**A**) and posterior (**P**) perineal hernias relative to the transverse perineal muscle.

Aetiological classification of perineal hernias include primary and secondary types. Primary congenital perineal hernias are rare and have been associated with failure of regression of the peritoneal cul de sac in the embryo.^2^ Primary acquired perineal hernias occur due to excessive stretching of the pelvic floor muscles including pregnancy, childbirth, constipation, obesity or chronic ascites.^3^ In our case, given the patient’s demographics, previous hysterectomy and anatomical location, we propose that the posterior perineal hernia is likely an acquired type. Secondary perineal hernias are incisional hernias related to previous surgeries, for example, abdominoperineal or urological procedures.^2^ Anatomical classification of perineal hernias is relative to the transverse perineal muscle, either anterior or posterior in location (Figure 4).

Approximately 100 cases of posterior perineal hernia have been reported in the literature. Most cases are female with only a few reported in males and paediatrics.^4,5^ Before the era of CT and MRI, diagnosis was either clinical or using barium enema.^3,6^ One case report in 2018 described the utility of 3D pelvic floor ultrasound.^7^ Most of the published cases have used CT scan for final diagnosis. To our knowledge, only three cases of perineal hernias have been reported in the literature using MRI as a diagnostic modality.^7–9^ There is no other published case on posterior perineal hernia secondary to only advanced age and previous hysterectomy as risk factors with MRI providing the diagnosis.

In symptomatic cases, common presentations are pain, incontinence or skin ulceration.^2^ A soft and reducible perineal, labial or gluteal mass may be palpable due to bowel, bladder, fat, lymph nodes or vessels in the hernial sac.^6,10^ Occasionally, intestinal obstruction or urinary dysfunction can occur. Strangulation is uncommon as the hernia neck is typically wide with an elastic muscular defect.^6^ There is no established ideal surgical approach for symptomatic cases. Surgical reduction and pelvic floor reconstruction are offered to suitable candidates as a definitive treatment option. Surgical approaches described in the literature include abdominal, transperineal or combined operation.^1^

In conclusion, this is a rare case of an elderly lady with an incidental finding of posterior Richter’s type perineal hernia. Initial CT findings raised the concern of a sinister locally advanced rectal mass. MRI pelvis provided a definitive diagnosis of perineal hernia but no sinister rectal pathology. Our case highlights the limitations of CT when findings are not definitive and the role of MRI as a superior problem-solving tool. Asymptomatic posterior perineal hernias can be managed conservatively and there is no established ideal surgical approach for symptomatic cases.

## Learning points

1. Perineal hernia can be an asymptomatic incidental finding on imaging.
2. CT imaging of perineal hernia can mimic a sinister rectal mass while MRI can better delineate the anatomy and confirm the diagnosis.
3. Understanding of key anatomical landmarks in pelvic floor anatomy and accurate interpretation of MRI imaging can prevent misdiagnosis as a pelvic mass.

## Informed consent statement

Written informed consent was obtained from the patient for publication of this case report, including accompanying images.
